# 基于铜死亡相关基因构建肺腺癌预后模型和药物敏感性分析

**DOI:** 10.3779/j.issn.1009-3419.2023.102.31

**Published:** 2023-08-20

**Authors:** Jihong SUN, Hanwen ZHANG, Haoran LIU, Yuqing DONG, Pingyu WANG

**Affiliations:** 264003 烟台，滨州医学院公共卫生与管理学院; School of Public Health and Management, Binzhou Medical College, Yantai 264003, China

**Keywords:** 肺肿瘤, 铜死亡, 预后模型, 免疫, 药物治疗, Lung neoplasms, Cuproptosis, Prognosis model, Immunity, Drug therapy

## Abstract

**背景与目的:**

肺癌是全球最常见的恶性肿瘤之一，目前肺癌筛查和治疗策略不断完善，但其5年生存率仍然很低，严重危害人类的健康。因此探索新的生物标志物，提供个体化治疗并改善患者预后至关重要。铜死亡是一种新发现的细胞死亡类型，是由于细胞内过量铜离子积累，最终导致细胞死亡，已有研究提示其与肺腺癌（lung adenocarcinoma, LUAD）的发生发展密切相关。本研究基于肿瘤基因组图谱（The Cancer Genome Atlas, TCGA）数据库，探究铜死亡相关基因（cuproptosis related gene, CRGs）与LUAD预后之间的关联，建立预后风险模型并分析CRGs与LUAD免疫细胞浸润之间的相互作用，为肺腺癌患者的治疗及预后提供参考。

**方法:**

从TCGA数据库中下载LUAD组织和癌旁或正常肺组织的RNA-seq数据，从基因型-组织表达资料库（Genotype-tissue Expression, GTEx）下载正常肺组织的RNA-seq数据，并从基因表达综合数据库（Gene Expression Omnibus, GEO）下载462例肺腺癌数据作为验证，采用单因素Cox和Lasso-Cox回归分析构建评估预后的风险评分模型，用受试者工作特征（receiver operating characteristic, ROC）曲线及校准曲线评价模型的预测能力。并进一步对高、低风险组进行免疫相关和药物敏感性分析。

**结果:**

共得到1656个CRGs，其中有1356个存在差异表达的CRGs，基于单因素Cox和Lasso-Cox回归分析筛选出13个CRGs构建预后风险模型，ROC曲线1、3、5年的曲线下面积（area under the curve, AUC）分别为0.749、0.740、0.689。进一步研究高、低风险组之间免疫相关功能和免疫检查点差异分析。高风险组对萨沃替尼、帕博西尼、阿糖胞苷等药物敏感性更高，更容易从免疫治疗中获益。

**结论:**

基于13个CRGs构建的风险模型具有较好的预后价值，可以辅助LUAD患者进行个体化治疗，为LUAD的治疗及预后研究提供了重要理论依据。

## Abstract (en)

**Background and objective:**

Lung cancer is one of the most common malignant tumors in the world, and the current lung cancer screening and treatment strategies are constantly improving, but its 5-year survival rate is still very low, which seriously endangers human health. Therefore, it is critical to explore new biomarkers to provide personalized treatment and improve the prognosis. Cuproptosis is a newly discovered type of cell death, which is due to the accumulation of excess copper ions in the cell, eventually leading to cell death, which has been suggested by studies to be closely related to the occurrence and development of lung adenocarcinoma (LUAD). Based on The Cancer Genome Atlas (TCGA) database, this study explored the association between cuproptosis-related genes (CRGs) and LUAD prognosis, established a prognostic risk model, and analyzed the interaction between CRGs and LUAD immune cell infiltration.

**Methods:**

The RNA-seq data of LUAD tissue and paracancerous or normal lung tissue were downloaded from the TCGA database; the RNA-seq data of normal lung tissue was downloaded from the Genotype-tissue Expression (GTEx) database, and the data of 462 lung adenocarcinoma cases were downloaded from the Gene Expression Omnibus repository (GEO) as verification. T the risk score model to assess prognosis was constructed by univariate Cox and Lasso-Cox regression analysis, and the predictive ability of the model was evaluated by receiver operating characteristic (ROC) curve and calibration curve. Immune-related and drug susceptibility analysis was further performed on high- and low-risk groups.

**Results:**

A total of 1656 CRGs and 1356 differentially expressed CRGs were obtained, and 13 CRGs were screened out based on univariate Cox and Lasso-Cox regression analysis to construct a prognostic risk model, and the area under the curves (AUCs) of ROC curves 1-, 3- and 5- year were 0.749, 0.740 and 0.689, respectively. Further study of immune-related functions and immune checkpoint differential analysis between high- and low-risk groups was done. High-risk groups were more sensitive to drugs such as Savolitinib, Palbociclib, and Cytarabine and were more likely to benefit from immunotherapy.

**Conclusion:**

The risk model constructed based on 13 CRGs has good prognostic value, which can assist LUAD patients in individualized treatment, and provides an important theoretical basis for the treatment and prognosis of LUAD.

肺癌是最常见的恶性肿瘤之一，也是全球癌症死亡的主要原因，近十几年来，我国肺癌的发病率与死亡率逐年攀升^[1]^。非小细胞肺癌（non-small cell lung cancer, NSCLC）是肺癌的重要病理类型，占所有肺癌病例的80%以上^[2]^，肺腺癌（lung adenocarcinoma, LUAD）是NSCLC的主要组织学亚型，约占所有肺癌病例的50%，并且其发病率正在逐年增加^[3]^。尽管目前LUAD的诊断技术和治疗手段已获得了很大的进步，但因为早期发现难、转移率高及长期缺乏系统诊断治疗等因素^[4]^，LUAD患者的预后仍然很差，5年生存率仅为10%- 20%^[5]^。所以，为LUAD患者识别可靠的预后生物标志物至关重要。

铜是人体必需的微量元素，也是各种酶促反应的必要辅助调节剂^[6]^。2022年，Tsvetkov等^[7]^提出了铜诱导的细胞死亡，称为铜死亡。有研究^[8]^发现铜死亡发生机制主要受到线粒体呼吸的影响，是铜直接与三羧酸循环的脂酰化成分相互作用，导致脂酰化蛋白质异常聚集和铁硫簇蛋白质缺失。最近的研究^[9⇓-11]^发现，铜死亡相关基因（cuproptosis related gene, CRGs）与人类癌症之间存在密切关系，但是，铜诱导的肿瘤死亡调控机制及在LUAD中的作用及临床意义尚不明确。

因此，本研究通过整合癌症基因组图谱（The Cancer Genome Atlas, TCGA）和基因型-组织表达资料库（Genotype-tissue Expression, GTEx）中LUAD转录组及临床数据，建立基于CRGs的LUAD患者预后风险评估模型，全面探索CRGs在LUAD中的分子机制及临床意义，并通过基因表达综合数据库（Gene Expression Omnibus, GEO）进行验证，为LUAD的预后和临床治疗提供有价值的参考。

## 1 资料与方法

### 1.1 数据来源

查阅最新文献获得19个CRGs^[12]^，分别为SLC31A1、PDHB、PDHA1、LIPT1、FDX1、DLD、DLST、DBT、LIAS、DLAT、GCSH、ATP7A、ATP7B、NFE2L2、NLRP3、LIPT2、MTF1、GLS、CDKN2A。从TCGA数据库（https://portal.gdc.cancer.gov/repository）获取LUAD的RNA-seq数据和临床信息，包括59个正常和541个LUAD样本的数据集。为弥补正常样本不足，从Xena公共数据中心（https://xenabrowser.net）GTEx数据库中下载288个正常样本。采用“limma”包将GTEx数据库中FPKM格式的RNA-seq数据转换成TPM格式，对数据进行批量归一化，将两个数据库结合统一为TPM格式。通过“TCGAbiolinks”R包获取TCGA体细胞突变数据，并下载拷贝数变异（copy number variation, CNV），绘制CNV景观^[13]^。

### 1.2 CRGs共表达及差异分析

在R语言中使用“limma”提取19个CRGs的表达量，再将CRGs与mRNAs进行共表达分析，过滤条件为|R|>0.5且P<0.001，识别CRGs^[14]^。之后识别正常组与LUAD组之间差异表达的CRGs，以|logFC|>2且FDR<0.05为过滤标准，并以火山图进行可视化^[15]^。

### 1.3 LUAD预后风险模型的构建和评估

单因素Cox回归分析筛选LUAD预后相关性CRGs，Lasso-Cox回归筛选独立预后的CRGs并构建预后模型，风险评分方程：Riskscore=（回归系数*mRNA_1_EXP）+（回归系数*mRNA_2_EXP）+......+（回归系数*mRNA_n_EXP）。根据风险评分中位数划分高、低风险组，通过风险模型热图和Kaplan-Meier生存曲线评估高低风险组总生存期（overall survival, OS）、无进展生存期（progression-free survival, PFS），绘制受试者工作特征（receiver operating characteristic, ROC）曲线以评估模型的预测效能。

### 1.4 列线图的构建与评估

利用“rms”包将预后相关的临床特征和风险评分进行单因素和多因素Cox回归分析，确定独立预后因素纳入列线图预测模型，并且利用校准曲线和决策分析（decision curve analysis, DCA）评估预测模型的准确性。

### 1.5 免疫相关分析

查阅文献收集到49个免疫检查点基因（immune checkpoint genes, ICGs）^[16]^，绘制箱式图评估LUAD高、低风险组患者之间的ICGs的差异。通过TIMER、CIBERSORT、CIBERSORTABS等7种计算方法分析免疫细胞在高、低风险组中的浸润情况，以热图的形式进行展示^[17]^。评估两组免疫功能、免疫检查点的差异，绘制箱线图展示。通过TIDE网站（http://tide.dfci.harvard.edu/）计算每例LUAD患者TIDE分数，比较两组差异。使用R软件的“ggpubr”包评估LUAD患者高、低风险组患者之间TIDE打分，以小提琴图进行可视化^[18]^。

### 1.6 免疫治疗潜在药物的筛选

使用“oncoPredict”包绘制箱线图，预测高、低风险组患者对常见抗癌药物的治疗反应，根据半数抑制浓度（half maximal inhibitory concentration, IC_50_）进行药物敏感性预测，评估LUAD高、低风险组患者对临床上经常使用癌症治疗药物敏感性的差异^[19]^。

### 1.7 GEO数据库提取表达数据

在PubMed主页面搜索栏中找到GEO datasets选项，进入GEO数据库，在搜索框中输入检索关键词“lung cancer”，筛选条件设置为“Homo sapiens"，下载芯片中矩阵及平台文件。采用R语言软件对数据进行归一化和矫正。

### 1.8 统计学处理

采用Perl 5.30.0版和R4.2.3版及相关的R程序包对数据进行统计分析。计量资料两组间比较采用Wilcoxon秩和检验。*P*<0.05为差异有统计学意义。

## 2 结果

### 2.1 LUAD中19个CRGs的景观

19 个CRGs在LUAD中的突变频率相对较低，其中NLRP3突变频率最高（12%），其次是CDKN2A和ATP7A（5%），FDX1、LIAS、LIPT1、SLC31A1、LIPT2、PDHB和GCSH在LUAD肿瘤组织中未显示突变（图1A）。图1B展示了CNV改变在染色体上的详细位置。CNV分析显示，CDKN2A、DBT、FDX1、DLAT、DLST、GCSH、PDHA1和PDHB的CNV丢失频率较高，而其他基因的CNV增益频率较高（图1C）。19个CRGs在LUAD患者中的异质性表达模型表明，它们可能在疾病的发展和进展中起到关键作用。

**图1:**
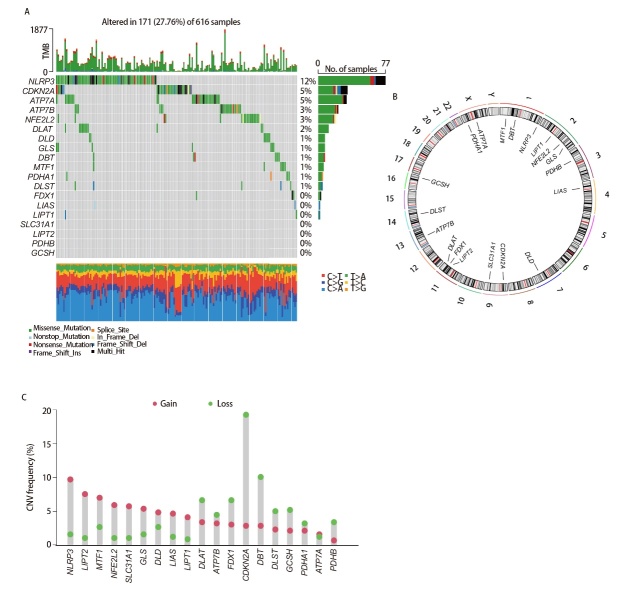
LUAD中19个CRGs的景观。A：CRGs在LUAD中的体细胞突变频率；B：LUAD中CRGs的CNV；C：CRGs在染色体上的位置。

### 2.2 LUAD中CRGs筛选结果

通过共表达分析共得到1656个CRGs，使用桑基图展示19个CRGs与1656个CRGs之间的共表达关系（图2A）。将GTEx资料库中288个正常肺组织的mRNAs表达数据与1656个CRGs取交集并进行差异分析，得到1356个差异表达的CRGs，结果以火山图的形式展示（图2B）。

**图2:**
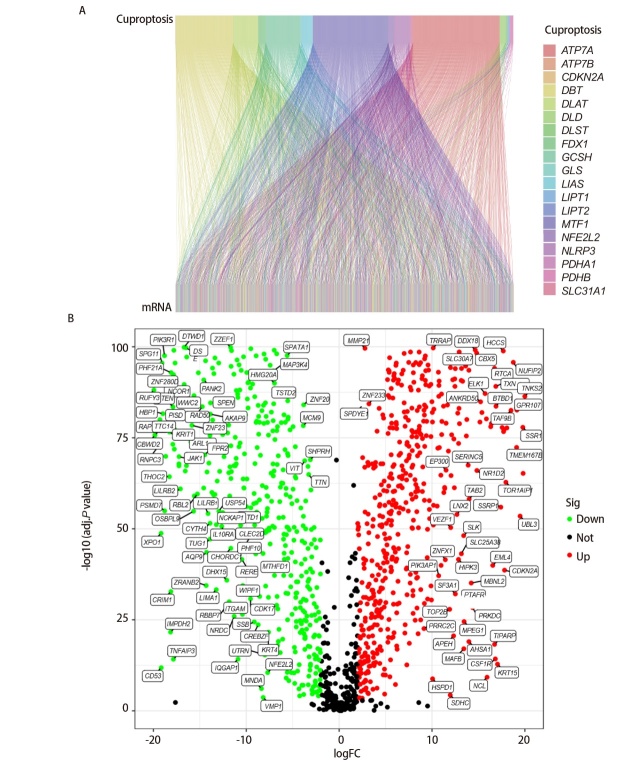
筛选差异表达的CRGs。A：19个CRGs和13个CRGs共表达关系的桑基图；B：差异表达的CRGs的火山图。

### 2.3 基于mRNA的LUAD预后模型的构建

剔除生存时间<30 d和临床数据缺失的样本，剩余472例患者纳入分析，使用单因素Cox回归分析筛选出284个具有预后作用的mRNA，再将上述mRNA进行Lasso-Cox回归分析，最终确定13个独立预后mRNA构建模型（图3A、图3B）。其中，MTMR12、TP63、CRYBG3、PDIK1L和UBR2为LUAD患者预后保护因素，CAMSAP2、ARL17A、MAML2、C5orf24、SLK、HNRNPR、NUP50和STX17为危险因素（表1），基于这13个mRNAs建立的LUAD患者预后风险评分方程为：风险评分=（1.207*ExpCAMSAP2）-（0.803*ExpMTMR12）-（0.574*ExpTP63）+（1.038*ExpARL17A）+（1.019*ExpMAML2）-（1.054*Exp CRYBG3）+（1.301*ExpC5orf24）+（0.855*ExpSLK）-（2.524*ExpPDIK1L）+（3.112*ExpHNRNPR）-（2.106*ExpUBR2）+（1.456*ExpNUP50）+（1.222*ExpSTX17）。绘制相关性热图将13个CRGs和查阅文献获得的19个CRGs之间的相关性进行可视化（图3C）。

**图3:**
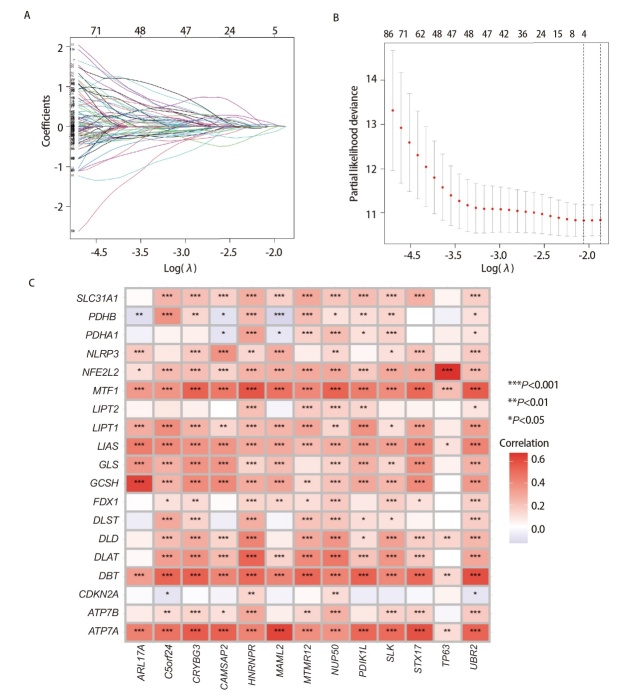
基于13个CRGs的LUAD患者构建风险模型。A：LASSO回归分析；B：λ选择图；C：13个CRGs和19个CRGs的相关性热图。

**表1:** 本研究通过数据分析鉴定的13种CRGs的多因素Cox回归分析

Variable	HR	95%CI	P
MTMR12	0.45	0.21-0.95	0.037
TP63	0.56	0.42-0.75	<0.001
CAMSAP2	3.34	1.36-8.21	0.008
ARL17A	2.83	1.50-5.34	0.001
MAML2	2.77	1.55-4.96	<0.001
CRYBG3	0.35	0.19-0.63	<0.001
C5orf24	3.67	1.58-8.51	0.002
SLK	2.35	1.23-4.49	0.010
PDIK1L	0.08	0.03-0.20	<0.001
HNRNPR	22.47	5.66-89.25	<0.001
UBR2	0.12	0.04-0.36	<0.001
NUP50	4.29	1.52-12.12	0.006
STX17	3.39	1.01-11.40	0.048

CRGs: cuproptosis related gene.

### 2.4 评估模型的预测价值

通过风险评分公式计算LUAD患者的风险评分，根据其中位数将所有LUAD患者划分为高、低风险组。Kaplan-Meier生存曲线分析结果表明，低风险组的OS和PFS更长（均P<0.001，图4A、图4B）。C-index曲线结果显示，风险评分的C-index从高到低依次为年龄、性别、种族、肿瘤原发灶-淋巴结-转移（tumor-node-metastasis, TNM）分期、吸烟史、原发肿瘤位置、放疗史（图4C）。应用预后模型预测LUAD患者1、3、5年生存率的曲线下面积（area under the curve, AUC）分别为0.749（95%CI: 0.656-0.826）、0.740（95%CI: 0.681-0.806）和 0.689（95%CI: 0.613-0.776）。绘制风险评分和临床特征的ROC曲线显示，风险评分对于预测LUAD患者生存率的准确性高于其他临床特征，说明我们构建的模型优于以上临床参数对LUAD患者的生存预测（图4D-图4F）。

**图4:**
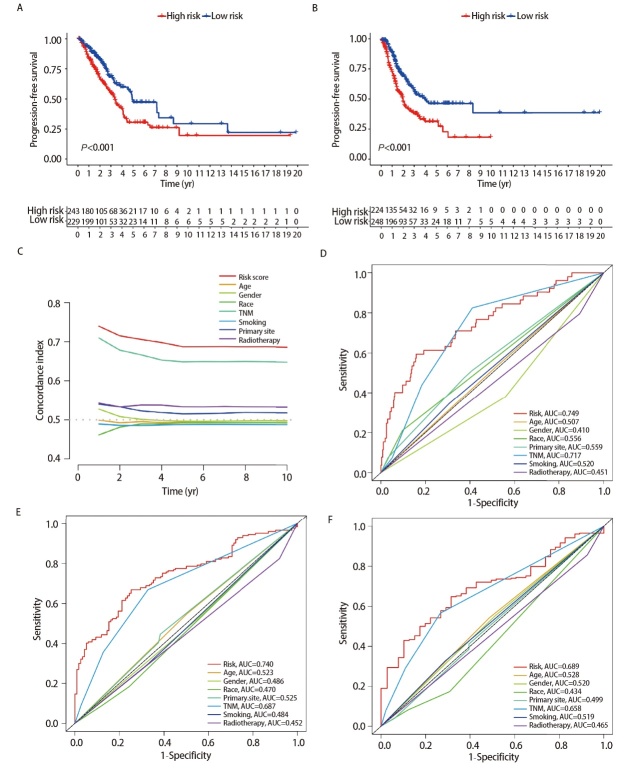
评估风险模型的预后价值。A：高、低风险组患者OS的生存曲线；B：高、低风险组患者PFS的生存曲线；C：风险评分与临床特征的C-index曲线；D-F：风险评分与其他临床特征的1、3、5年ROC曲线。

### 2.5 GEO数据集中CRGs的验证

为了验证以上CRGs的预后价值，通过外部数据集GSE68465其中包括462例肺腺癌样本，绘制Kaplan-Meier生存曲线显示，高风险组患者预后比低风险组差（P<0.001）。根据ROC曲线AUC得出，CRGs预测患者1、3、5年的生存率AUC分别为0.645、0.639、0.670（图5）。

**图5:**
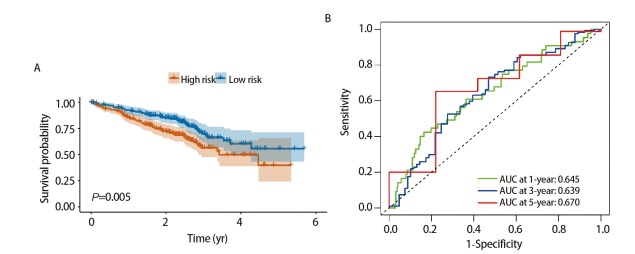
GEO队列中LUAD中CRGs的预后特征。A：GEO队列中患者OS的生存曲线；B：GEO队列中患者的ROC曲线。

### 2.6 评估TCGA-LUAD患者的独立预后特征

为了评估LUAD患者预后的独立因素，将风险评分、年龄、性别、种族、肿瘤原发位置、TNM分期、吸烟史和放疗史全部纳入单因素及多因素Cox回归分析，将多种临床特征之间的关联进行综合考虑，结果显示TNM分期为II期（HR=2.22, 95%CI: 1.53-3.23, P<0.001）、III期（HR=3.09, 95%CI: 2.05-4.64, P<0.001）、IV期（HR=2.82, 95%CI: 1.54-5.15, P<0.001）以及风险评分为高风险组（HR=2.90, 95%CI: 2.08-4.03, P<0.001）是LUAD患者预后危险影响因素（表2）。

**表2:** LUAD预后的多因素Cox回归分析

Variable	*n*	HR	95%CI	*P*
Age				
≤65 yr	226	Reference		
>65 yr	246	1.10	0.81-1.50	0.600
Gender				
Male	218	Reference		
Female	254	0.95	0.70-1.30	0.800
Smoking				
Yes	325	Reference		
No	147	1.16	0.83-1.62	0.400
Radiation				
Yes	56	Reference		
No/Unknown	416	0.76	0.49-1.17	0.200
Race				
White	370	Reference		
Blake	49	0.91	0.53-1.55	0.700
Unknown	53	1.19	0.72-1.98	0.500
Primary site				
Upper lobe of lung	272	Reference		
Middle lobe of lung	20	1.18	0.47-2.97	0.700
Lower lobe of lung	162	1.09	0.79-1.51	0.600
Other	18	0.76	0.36-1.60	0.500
TNM				
Ⅰ	256	Reference		
Ⅱ	114	2.22	1.53-3.23	<0.001
Ⅲ	77	3.09	2.05-4.64	<0.001
Ⅳ	25	2.82	1.54-5.15	<0.001
Risk				
Low risk	248	Reference		
High risk	224	2.90	2.08-4.03	<0.001

### 2.7 构建并评估TCGA-LUAD列线图预后模型

将年龄、性别、TNM分期、风险评分纳入构建LUAD患者列线图模型（图6A）。列线图预测LUAD患者1、3和5年生存率的AUC分别为0.805、0.803和0.752。与风险评分模型相比，列线图对LUAD患者生存率的预测更加准确（图6B）。校准曲线显示列线图预测患者1、3、5年生存率曲线与实际生存率曲线吻合度较高（图6C），表明模型准确度高、稳定性强。DCA分析显示该模型能够更准确地预测LUAD患者的预后，可为临床管理和决策提供参考（图6D）。

**图6:**
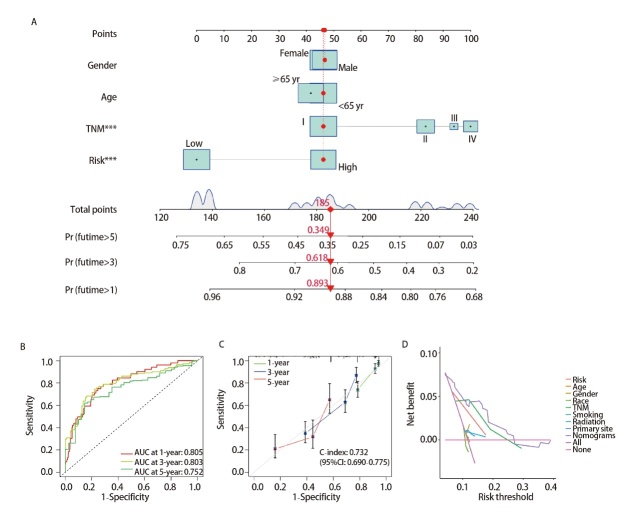
构建及验证LUAD的列线图预后模型。A：LUAD患者列线图模型；B：预测LUAD患者的1、3、5年生存率的AUC值；C：列线图的校准曲线预测LUAD患者的1、3、5年生存率；D：DCA评估模型的临床疗效。

### 2.8 风险评分与免疫相关分析

查阅文献共获得49个ICGs（图7A），差异分析结果显示，49个ICGs中有24个ICGs在高、低风险组患者中的表达量存在差异（P<0.05），且只有CD276在高风险组中呈高表达状态。表明风险评分模型与ICGs具有较强的相关性（图7B）。通过TIDE网站（http://tide.dfci.harvard.edu/）对LUAD高、低风险组患者免疫治疗反应进行预测，结果发现，高风险组患者的得分高于低风险组患者（P<0.001，图7C），说明低风险组患者发生免疫逃逸的可能性较低，对免疫治疗的效果可能会更好。采用TIMER、CIBERSORT、CIBERSORT-ABS、QUANTISEQ、MCPCOUNTER、XCELL和EPIC等7种方法评估免疫细胞相关性，发现多种免疫细胞在低风险组表达较高，如记忆B细胞、CD8^+ ^T细胞、记忆静息CD4^+ ^T细胞等，差异均有统计学意义（P<0.05，图8A），即低风险组患者表现出强免疫状态。在免疫功能差异分析的箱线图中，低风险组免疫功能的得分更高，说明低风险组免疫功能更加活跃，例如人类白细胞抗原（human leukocyte antigen, HLA）功能等（P<0.001，图8B）。

**图7:**
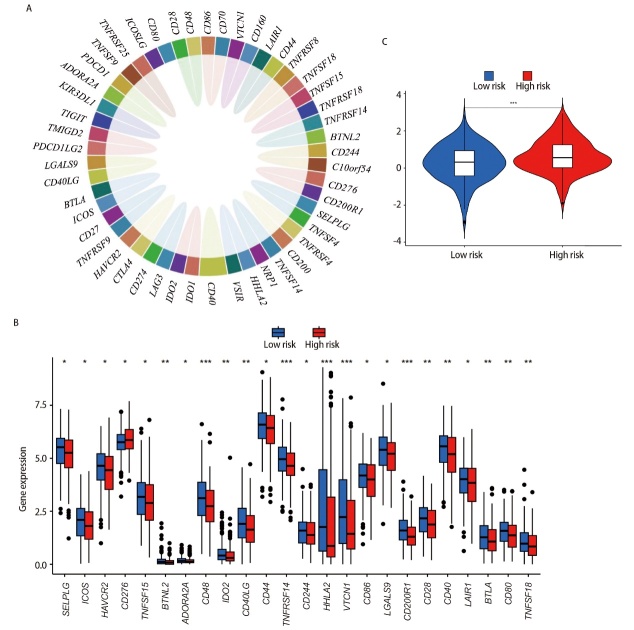
高、低风险组免疫微环境分析。A：49个免疫相关基因；B：免疫检查点基因表达差异分析；C：高、低风险组患者免疫逃逸和免疫治疗。*：P<0.05；**：P<0.01；***：P<0.001。

**图8:**
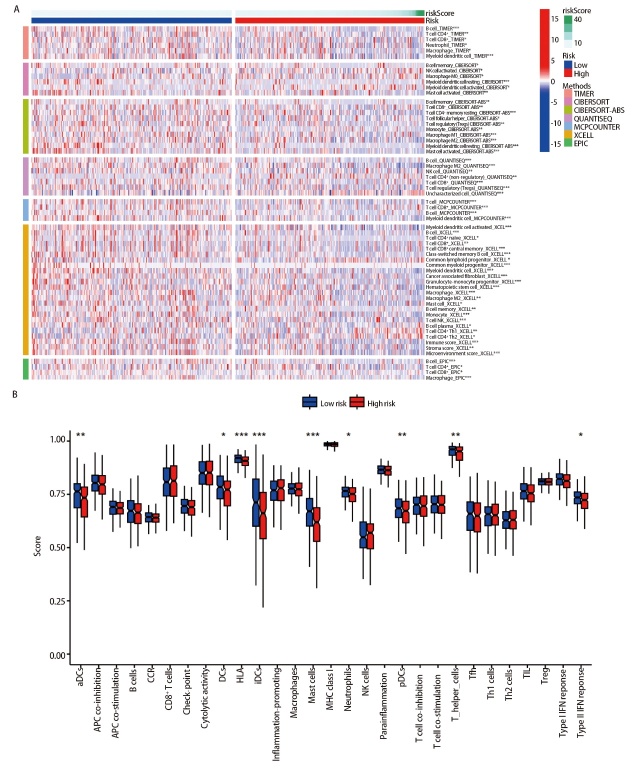
高、低风险组免疫功能和免疫细胞分析。A：高、低风险组患者免疫细胞相关性分析；B：高、低风险组免疫功能差异分析。*：P<0.05；**：P<0.01；***：P<0.001。

### 2.9 潜在药物敏感性分析

使用“oncoPredict”包筛选LUAD患者对198种抗肿瘤药物的IC_50_值。其中横坐标代表LUAD高、低风险组患者，纵坐标代表IC_50_值，结果发现高风险组患者的IC_50_值较低，说明高风险组患者对萨沃替尼、帕博西尼、阿糖胞苷、拉唑帕尼、福替尼、MDM2抑制剂等药物敏感性更高（P<0.01，图9），更有利于接受药物治疗。

**图9:**
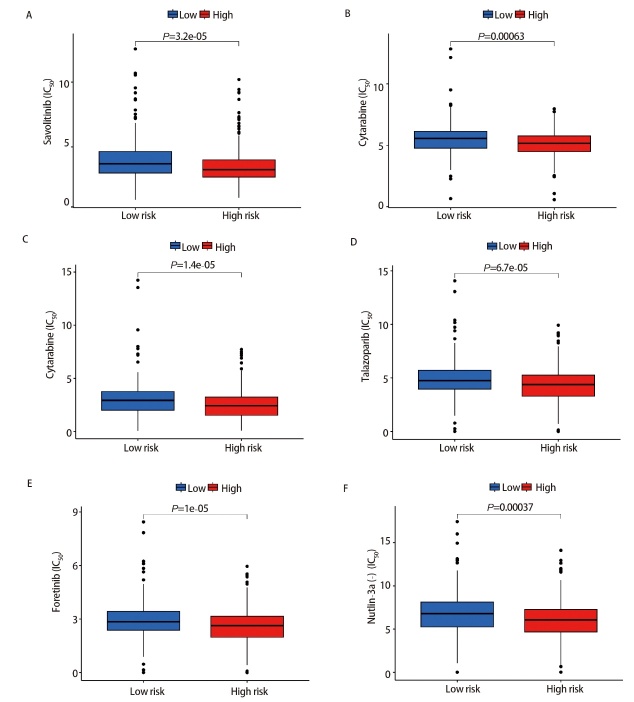
高、低风险组之间药物敏感性差异分析。A：萨沃替尼；B：帕博西尼；C：阿糖胞苷；D：拉唑帕尼；E：福替尼；F：MDM2抑制剂。

## 3 讨论

肺癌是世界上最常见的癌症类型，尽管肿瘤筛查和治疗手段不断完善，但由于肺癌的异质性和侵袭性，其5年生存率仍然很低，改善患者的不良预后情况迫在眉睫^[20]^。铜死亡是最新发现的细胞死亡类型，是由于细胞内过量铜离子积累引发的，有研究^[21]^已经确定其与LUAD的发生和发展密切相关。

本研究通过整合TCGA和GTEx数据库，构建了一个由13个CRGs构成的LUAD预后风险模型，其中MTMR12、TP63、CRYBG3、PDIK1L和UBR2为LUAD患者预后保护因素，CAMSAP2、ARL17A、MAML2、C5orf24、SLK、HNRNPR、NUP50和STX17为危险因素，其中许多基因已经被鉴定出来了。例如，TP63是抑癌基因TP53的家族成员，该基因家族对细胞分化和应激反应至关重要，TP63的多态性变异与LUAD的增殖与发生密切相关。CRYBG3过量表达可以抑制肺癌细胞增殖并促进细胞死亡，作用机制是通过CRYBG3直接与G-肌动蛋白结合来阻断细胞分裂，可作为抑制剂抑制体内肿瘤发展。NSCLC的发展过程可以通过敲低circSOD2的表达，然后上调CAMSAP2和miR-2355-5p来逆转。CAMSAP2是miR-2355-5p的靶点，其在NSCLC细胞和组织中呈高表达。MAML2基因异位通过破坏正常细胞生长和分化，对原发性肺黏液表皮样癌患者生存率产生显著影响。在LUAD中HNRNPR的蛋白表达水平远高于正常组织，晚期患者HNRNPR表达显著升高，提示HNRNPR可能促进癌细胞的增殖、迁移和侵袭能力^[22⇓⇓⇓-26]^。总之，这些基因在肺癌发生发展过程中起重要作用，进一步研究可能会为LUAD患者提供新的治疗策略。

先前研究^[27]^构建的基于6个CRGs的LUAD预后风险模型中患者1、3、5年生存率的AUC分别为0.639、0.605、0.576，而本研究中应用生存分析、ROC、校准曲线评估了模型的准确性。结果显示低风险组患者的OS和PFS高于高风险组患者且预测LUAD患者1、3和5年生存率的AUC分别为0.749、0.740和0.689。因此与以往LUAD中发表的CRGs风险模型相比，本研究构建的模型更加精确可靠，对LUAD患者预后预测效果更好。

免疫功能与免疫细胞浸润在肿瘤发生进展中起着不可或缺的作用^[28]^。免疫功能分析显示，低风险组患者更多地参与浆细胞样树突细胞、辅助性T细胞、人类白细胞抗原、II型干扰素反应等免疫功能。我们采用七种方法评估免疫细胞相关性，结果发现记忆B细胞、CD8^+ ^T细胞、记忆静息CD4^+ ^T细胞等免疫浸润细胞在低风险组高表达。ICGs差异分析显示，只有CD276在高风险组中呈高表达状态。CD276在恶性肿瘤细胞增殖、侵袭、迁移免疫逃逸密切相关，由于CD276在多种肿瘤中的表达水平升高，有研究^[29]^使用CD276作为肿瘤基因治疗和单克隆抗体治疗的靶标。高风险组患者的肿瘤微环境（tumor microenvironment, TME）免疫浸润水平较低，两组免疫功能存在差异。说明铜死亡与肿瘤免疫相互影响，高风险组中免疫功能可能受到抑制。

本研究继续探索了不同风险组对免疫治疗的反应，其药物敏感性在两个组别中差异有统计学意义，并且高风险组患者更加敏感。有研究^[30]^表明萨沃替尼是一种选择性间质上皮转化因子（mesenchymal-epithelial transition, MET）抑制剂，可应用于多种恶性肿瘤。本研究中高风险组患者对以上药物的IC_50_值较低，更适合以上药物治疗，可为临床治疗中的药物选择提供了理论支持。

本研究基于公共数据库构建了LUAD中CRGs的预后模型，并验证其准确性和应用价值，该模型能更好地预测患者的预后。但本研究仍存在一定的不足。首先，我们的数据是从公共数据库中获取的，因此后续还需要结合临床样本实施分子生物学实验进一步进行验证。其次，我们通过生物信息学挑选出的基因，其作用机制还不清楚，有待进一步研究。

**Competing interests**

The authors declare that they have no competing interests.
